# The Relation between Caffeine Consumption and Endometriosis: An Updated Systematic Review and Meta-Analysis

**DOI:** 10.3390/nu13103457

**Published:** 2021-09-29

**Authors:** Konstantinos S. Kechagias, Konstantinos Katsikas Triantafyllidis, Margarita Kyriakidou, Panagiotis Giannos, Ilkka Kalliala, Areti Angeliki Veroniki, Maria Paraskevaidi, Maria Kyrgiou

**Affiliations:** 1 Department of Metabolism, Digestion and Reproduction, Faculty of Medicine, Imperial College London, London W12 0NN, UK; i.kalliala@imperial.ac.uk (I.K.); a.veroniki@imperial.ac.uk (A.A.V.); m.paraskevaidi@imperial.ac.uk (M.P.); m.kyrgiou@imperial.ac.uk (M.K.); 2 Department of Dietetics, West Suffolk Hospital NHS Foundation Trust, Bury St Edmunds, Bury Saint Edmunds IP33 2QZ, UK; k.katsikas-triantafyllidis18@alumni.imperial.ac.uk; 3 Society of Meta-Research and Biomedical Innovation, London W12 0BZ, UK; panagiotis.giannos19@imperial.ac.uk; 4 Department of Applied Mathematical and Physical Sciences, National Technical University of Athens, 15773 Athens, Greece; m.kyriakidou@aibs.gr; 5 Department of Life Sciences, Faculty of Natural Sciences, Imperial College London, London SW7 2AZ, UK; 6 Department of Obstetrics and Gynaecology, Helsinki University and University Hospital Helsinki, 00014 Helsinki, Finland; 7 Knowledge Translation Program, Li Ka Shing Knowledge Institute, St. Michael’s Hospital, Toronto, ON M5B 1W8, Canada; 8 West London Gynaecological Cancer Centre, Imperial College NHS Trust, London W12 0HS, UK

**Keywords:** caffeine, coffee, caffeine-containing beverages, endometriosis, environmental factors, meta-analysis, review

## Abstract

While the contributing factors leading to endometriosis remain unclear, its clinical heterogeneity suggests a multifactorial causal background. Amongst others, caffeine has been studied extensively during the last decade as a putative contributing factor. In this systematic review and meta-analysis, we provide an overview/critical appraisal of studies that report on the association between caffeine consumption and the presence of endometriosis. In our search strategy, we screened PubMed and Scopus for human studies examining the above association. The main outcome was the relative risk of endometriosis in caffeine users versus women consuming little or no caffeine (<100 mg/day). Subgroup analyses were conducted for different levels of caffeine intake: high (>300 mg/day) or moderate (100–300 mg/day). Ten studies were included in the meta-analysis (five cohort and five case-control studies). No statistically significant association was observed between overall caffeine consumption and risk for endometriosis (RR 1.12, 95% confidence interval (CI) 0.97–1.28, I^2^ = 70%) when compared to little or no (<100 mg/day) caffeine intake. When stratified according to level of consumption, high intake was associated with increased risk of endometriosis (RR 1.30, 95%CI 1.04–1.63, I^2^ = 56%), whereas moderate intake did not reach nominal statistical significance (RR 1.18, 95%CI 0.99–1.40, I^2^ = 37%). In conclusion, caffeine consumption does not appear to be associated with increased risk for endometriosis. However, further research is needed to elucidate the potential dose-dependent link between caffeine and endometriosis or the probable role of caffeine intake as a measurement of other unidentified biases.

## 1. Introduction

Endometriosis is a common disorder defined as the presence of endometrial tissue (glandular cells and stroma) outside the uterine cavity [1]. The most common sites of endometriosis include the pelvic peritoneum, the ovaries, and the uterosacral ligaments. Women with endometriosis may be asymptomatic or suffer from subfertility, pelvic pain, and dyspareunia [2]. The disease affects 2–10% of women of reproductive age and 30–50% of the female population in general, but the actual prevalence is unknown, because the diagnosis is only established by surgery [3].

While the definitive cause of endometriosis constitutes a matter of debate, its clinical heterogeneity suggests a multifactorial causal background that consists of both genetic and environmental factors [4]. During the last two decades, several studies have correlated endometriosis with modifiable risk factors, such as food intake and lifestyle habits, given their potential influence on hormonal levels, immune response, and inflammatory activity [5,6,7].

Caffeine, one of the most widely used pharmacologically active substances worldwide, has been studied extensively as a potential contributing factor linked with the development of hormone-dependent conditions [8]. This theory stems from the fact that caffeine affects the levels of steroid hormones, the production of the sex hormone-binding globulin in the liver, and the conversion of androgens to estrogens by altering aromatase function [9,10]. Based on the above, many scientists hypothesised that these hormonal changes could lead, or partially act as contributing factors, to the development of endometriosis.

### Objective

The aim of this systematic review and meta-analysis was to provide an updated overview of the available literature from human studies on the association between caffeine consumption and endometriosis and to further stratify this according to the level of caffeine consumption.

## 2. Methods

The systematic review was designed and reported based on the PRISMA guidelines, and it was registered in the open science framework (registration doi:10.17605/OSF.IO/UK5JX).

### 2.1. Literature Search

We searched PubMed and Scopus for articles published from inception until January 2021. Two authors (K.K.S. and K.K.T.) searched all databases independently. There were no language and geographic region restrictions. The terms used for the PubMed search were: (coffee OR caffeine OR caffeine beverages OR diet OR tea) AND (endometriosis). In Scopus, search was limited to ‘articles’ regarding the study type, using the same terms. Reference lists of relevant reviews and articles selected for inclusion were additionally manually searched. Abstracts submitted in conferences and other non-peer-reviewed sources were not eligible for inclusion. Discrepancies in the literature search process were discussed and resolved by M.K.

### 2.2. Eligibility Criteria

We conducted a systematic review that included studies that described the association between caffeine consumption/exposure and endometriosis irrespective of dose consumed and study design (observational studies, case-series, and randomized controlled trials). However, only studies reporting on results from a comparison group were included in the meta-analysis. Additionally, only studies providing data on caffeine consumption for both endometriosis cases and healthy individuals were considered eligible for the meta-analysis.

Studies on in vitro and experimental animal models as well as studies reporting on scar endometriosis were not eligible for inclusion in the analysis. For studies that examined different caffeine-containing beverages (e.g., coffee, tea, cola, chocolate), the overall caffeine intake was retrieved as estimated in the original study. For studies that provided data on a monthly caffeine intake, the consumption was converted to daily intake dividing by 30. For studies measuring caffeine consumption using cups per day, it was assumed that one cup corresponds to 100 mg of caffeine [11,12].

### 2.3. Data Extraction and Risk of Bias

Two authors (K.S.K. and K.T.K.) extracted data independently including the name of the first author, date of publication, country of origin, study design, number of subjects, age of participants, site of endometriosis for cases, effect sizes (e.g., risk ratios (RR), hazard ratios (HR), or odds ratios (OR)) of endometriosis, corresponding uncertainty measures such as 95% confidence intervals (CI) for coffee intake categories, raw data in the form of 2 × 2 tables as well as adjusted and unadjusted effect sizes if they were available.

The Methodological Index for Non-Randomised Studies (MINORS) was used for the risk of bias assessment of observational studies [13]. MINORS is a valid instrument designed to assess the methodological quality of non-randomised studies, whether comparative or non-comparative. It is a 12-item tool (maximum of 24 points) that among others assesses the statistical methodology, the inclusion and exclusion criteria, the aim, and the control group of the included studies. MINORS ≤ 9 were considered as high risk of bias, while MINORS between 10 and 14 were considered as moderate risk of bias [13].

### 2.4. Data Synthesis

Our primary analysis reported on the association between overall caffeine consumption compared to little or no caffeine (<100 mg/day) and pelvic endometriosis. We performed further subgroup analyses separately for high (>300 mg/day) and moderate (100–300 mg/day) versus little or no caffeine (<100 mg/day). In these, we only included studies providing quantities in mg. Furthermore, we performed sensitivity analyses based on the country of origin, the study design, the type of diagnosis (surgical vs. medical), the risk of bias of the included studies, and considering coffee as the only source of caffeine.

Study effect sizes were combined along with corresponding 95% CIs under the random-effects meta-analysis model and the Mantel–Haenszel method [14]. Statistical heterogeneity was assessed by using the χ^2^ test (*p* < 0.10 to indicate statistically significant heterogeneity) and I^2^ (to quantify the degree of heterogeneity) [15,16]. I^2^ from 30% to 49% was defined as moderate heterogeneity and 50% or more was defined as high heterogeneity for the data. We also estimated the 95% prediction interval, which further accounts for between-study heterogeneity and evaluates the uncertainty for the effect that would be expected if a new study addresses that same association in the future. The Der Simonian and Laird estimator was used to estimate the between-study variance [17,18]. Visual inspection of the funnel plot and the Egger’s test were used to assess for small-study effects [19].

The meta-analysis was performed using RevMan (Review Manager) Web in the online platform provided for Cochrane intervention reviews (RevMan, Copenhagen: The Nordic Cochrane Centre, the Cochrane Collaboration, 2008). The forest plots were drawn using R software (version 3.3.1, R Foundation for Statistical Computing, Vienna, Austria).

## 3. Results

### 3.1. Study Characteristics

**Figure 1:**
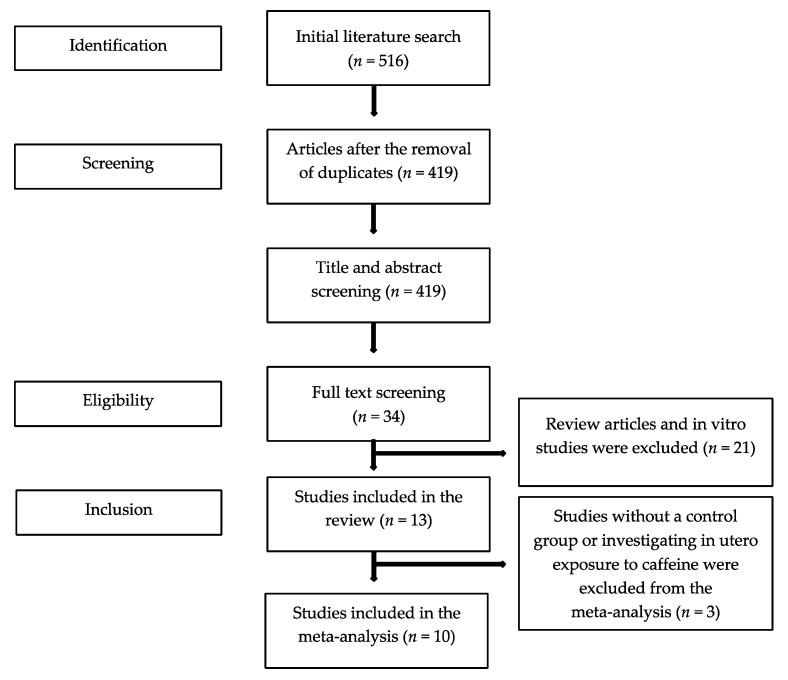
Flowchart of the literature search process.

The initial literature search yielded 516 publications. After the exclusion of duplicates, 34 full texts were screened, and 13 studies were found eligible for the systematic review (Figure 1). All 13 studies were observational; seven were cohort [20,21,22,23,24,25,26] and six were case-control [27,28,29,30,31,32] studies. The majority of the studies (10/13) explored caffeine as part of a number of risk factors that associated with endometriosis in women with and without disease. In six studies, caffeine consumption was based on the intake of caffeine containing beverages (e.g., coffee, tea, cola, chocolate), in four studies, caffeine consumption was based on coffee intake only, and in three studies, beverage type was not defined. In 10 studies, endometriosis was diagnosed surgically (laparoscopy or laparotomy), in two, endometriosis was defined clinically or using imaging [23,24], and one study [26] did not describe the method of diagnosis. Nine studies were conducted in America, three were conducted in Europe, and one was conducted in Asia. Of the 13 studies, one provided data only for women with endometriosis [20], one examined in utero exposure to caffeine [23], and one reported on the risk for scar endometriosis [31]. Consequently, 10 studies were finally included in the meta-analysis (Table 1).

### 3.2. Risk of Bias

The MINORS score for the 10 observational studies that were included in the analysis ranged from seven to 15, suggesting high to moderate risk of bias. Five studies were considered as high risk of bias (MINORS < 9), and the remaining five were considered as moderate risk of bias (MINORS between 9 and 15). The follow-up period was inadequate in nine out of 10 studies (Table 2).

### 3.3. Caffeine and Endometriosis Analysis

**Figure 2:**
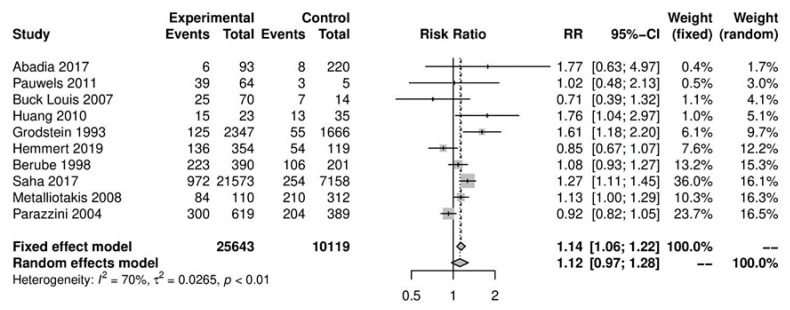
Main analysis of events (endometriosis cases) in caffeine intake group (>100 mg/day) versus little or no caffeine intake (<100 mg/day).

In the primary analysis, overall caffeine intake (>100 mg/day) increased the risk of endometriosis by 12%, but this difference was not statistically significant (10 studies; 38,601 participants; RR 1.12, 95% CI 0.97–1.28; I^2^ = 70%) (Figure 2) when compared to little or no caffeine use (<100 mg/day). Substantial heterogeneity was observed.

**Figure 3:**
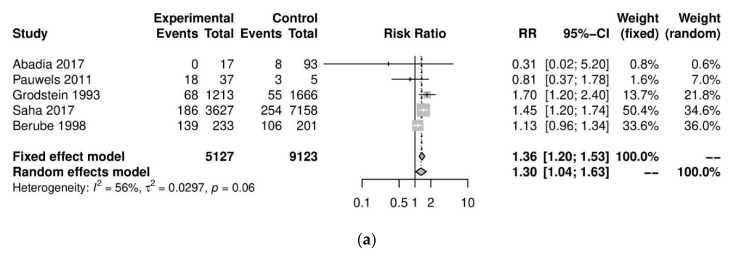

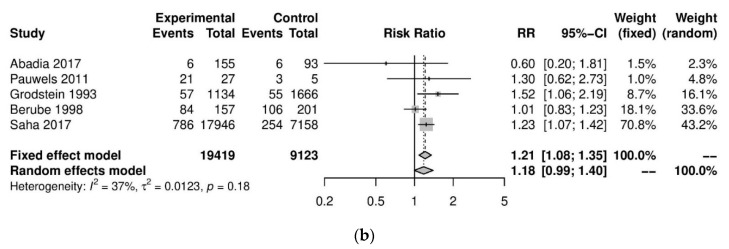
Analysis of (**a**) high caffeine intake (>300 mg/day) versus little or no caffeine (<100 mg/day) and (**b**) moderate caffeine intake (200–300 mg/day) versus little or no caffeine (<100 mg/day).

We performed further subgroup analyses to stratify according to the level of caffeine intake (Figure 3). High caffeine consumption (>300 mg/day) significantly increased the risk of endometriosis when compared to little or no caffeine (<100 mg/day) (five studies; 15,085 participants; RR 1.30, 95% CI 1.04–1.63; I^2^ = 56%). Moderate caffeine intake (100–300 mg/day) also increased the risk of endometriosis but the difference did not reach significance (five studies, 29,920 participants, RR 1.18, 95%CI 0.99–1.40, I^2^ = 37%). However, 95% prediction intervals failed to exclude the null value (high caffeine intake 95% PI (0.77–2.22); moderate caffeine intake 95% PI (0.85–1.63)), which was possibly due to the high heterogeneity observed.

We performed a series of sensitivity analyses for the primary analysis which showed consistent results and did not reduce the high heterogeneity between studies. Sensitivity analyses on the risk of endometriosis in women with high/moderate versus little or no caffeine consumption were conducted based on country (Americas: RR 1.10, 95% CI 0.92–1.31, I^2^ = %; Europe: RR 1.07, 95% CI 0.8–1.44, I^2^ = 85%); study design (case-control studies: RR 1.19, 95% CI 0.94–1.5, I^2^ = 75%; cohort studies: RR 1.07, 95% CI 0.88–1.3, I^2^ = 67%); type of diagnosis (surgical diagnosis: RR 1.08, 95% CI 0.93–1.25, I^2^ = 67%); MINORS score of the study (moderate risk of bias: RR 1.04 95% CI 0.82–1.32, I^2^ = 82%), and coffee as the only caffeine source (RR 1.15 95%, CI 0.95–1.39, I^2^ = 81%). No evidence of small-study effects was observed in the main analysis (Egger’s *p* = 0.97).

## 4. Discussion

### 4.1. Main Findings

In our analysis, the consumption of caffeine was not associated with increased risk of endometriosis when compared to women consuming little or no caffeine. The primary analysis irrespective of the level of caffeine and the analysis on moderate caffeine intake (100–300 mg/day) did not reach statistical significance. However, intake of higher quantities (>300 mg per day) reached statistical significance. Additionally, high risk of bias and heterogeneity among studies were observed.

### 4.2. Findings in Context of the Literature

Clinical studies examining the association of modifiable risk factors, such as caffeine, with endometriosis are scarce in the literature, and their results are equivocal. Two studies from the mid-1990s, conducted in the USA and Canada, showed a statistically significant increased risk of endometriosis among patients who consumed caffeine-containing beverages [27,28], However, case-control studies published the following years reached opposing conclusion describing no association [22,25,29,30,32]. A recent well-designed Swedish study with almost 30,000 participants reported a positive association of endometriosis with daily consumption of coffee in the analysis of unadjusted data, but statistical significance was not reached upon adjustment [24]. Therefore, the impact of caffeine on endometriosis and other hormone-dependent conditions still constitutes a matter of debate.

Our results are partially in agreement with a former meta-analysis, which included articles published before January 2013, and concluded that caffeine consumption was overall not significantly associated with increased risk of endometriosis [33]. However, the possible association with high caffeine intake was not proposed. This meta-analysis included only eight studies (1407 participants), and one of the included studies provided data only for patients with endometriosis, which could potentially lead to inaccurate results. The authors included no risk of bias assessment, and subgroup analyses according to the level of caffeine consumption were not defined accurately based on mg/day. Our analysis included only studies with a comparison group with rigorous assessment of risk of bias, with the addition of three new studies and over 38,600 participants.

Studies exploring in utero exposure to caffeine were not included in the meta-analysis. These have reported conflicting results with some proposing no association or even a reduction in the risk of endometriosis [21,23]. The impact of caffeine on the early stages of embryo development is far from established.

Many putative mechanisms have been proposed to explain the potential link between caffeine consumption and endometriosis. Some of these present conflicting suggested mechanisms, and the pathophysiology remains largely unclear. Caffeine inhibits the activity of aromatase, which is a key enzyme for the peripheral conversion of androgens to estrogens, and as a result, it affects the level of estrogens [34,35]. Moreover, it affects the hepatic function, increases the secretion of sex hormone-binding globulin (SHGB), and as a consequence decreases the bioavailability of steroid hormones [36]. However, this effect has been shown to be lost after 8 weeks following exposure and has also been noticed after the consumption of decaffeinated beverages [10]. The above alterations could create a hormonal milieu that potentially contributes to the pathogenesis and presentation of the disease. These hormonal alterations may constitute an exogenous factor that acts synergistically with phenomena such as coelomic metaplasia, proliferation of progenitor stem cells, or retrograde menstruation of endometrial cells, all of which are thought to lead to the implantation and proliferation of ectopic endometrial cells [37].

### 4.3. Strengths and Limitations

This is the first systematic review that included studies reporting data from women with caffeine intake versus women with little or no caffeine intake and documented that caffeine, particularly if consumed in large amounts, can potentially be associated with increased risk of endometriosis and calls for further research in the field. Additionally, we applied rigorous assessment of bias using MINORS, which is a well-established and accurate risk of bias tool for observational studies, in an attempt to overcome the low quality of available studies related to this topic [38].

However, the results should be interpreted with caution. We could not find randomised controlled trials on the subject and adjusted data from the observational studies were lacking. Specifically, covariates that could potentially influence caffeine consumption and endometriosis such as diet type (vegetables, whole grain, processed meat), physical activity, weight, and gravidity were not considered as potential confounders across all the included studies. Therefore, an analysis of confounders was not feasible.

The overall quality of data was low based on the low median MINORS score. This could be partially attributed to the inclusion of studies that were not designed to answer the primary outcome of this systematic review. The observed outcomes of the meta-analysis were also limited by the small number of included studies, the high heterogeneity observed, and the fact that the majority of the patients included in the main and subgroup analyses came from a single study. Many studies, which evaluated the association of diet with endometriosis, could not be included because specific data regarding caffeine consumption quantities were not available. The high diversity between the methods of caffeine intake assessment used in the studies and the inclusion of different caffeine containing beverages can also influence the results. Data regarding co-morbidities of patients, such as the presence of infertility, was not provided for the majority of the included studies. As a result, classification of participants based on fertility status was not feasible. In addition, the attempt to form subgroups for specific sites and severity of endometriosis was not possible due to the lack of relevant data.

Although our analysis showed evidence of association between high caffeine consumption and endometriosis, this association may not be causal but an indication of bias. For instance, caffeine consumption has been previously associated with decreased rates of conception [19]. Women presenting with infertility are then more likely to undergo investigations and be given the diagnosis of endometriosis. Therefore, caffeine consumption may not cause or contribute to endometriosis, but it may be more common in the subgroup of women that are diagnosed with the disease.

One way to tackle the above limitations is to design Mendelian Randomisation studies [39]. Mendelian randomisation is a method of using the association of variation in genes with modifiable exposures, such as caffeine consumption, to examine their causal effect on disease outcomes in observational studies. While these methods are still in their infancy, they can be used as a valuable strategy to examine causality in complex biological networks in the future [40].

## 5. Conclusions

Our meta-analysis suggests that caffeine consumption does not seem to be associated with increased risk of endometriosis. However, high quantities of caffeine intake (>300 mg/day) may be possibly associated with the disease. Results should be interpreted with caution due to the high risk of bias and heterogeneity among studies. Although our findings present an association of high caffeine intake with endometriosis, they do not infer causality, as caffeine consumption could potentially act as a measure of other unidentified confounding factors. Well-designed large clinical studies and Mendelian Randomisation approaches are required to elucidate this potential relation and determine the exact role of caffeine in the pathophysiology of endometriosis.

## Figures and Tables

**Table 1:** Characteristics of the included studies.

Author, Year, Country	Study Design	Age Range	N of Participants Consuming Moderate or High Caffeine **	N of Participants Consuming Little or No Caffeine ***	Follow-Up Period	Endometriosis Group	Comparator Group	Estimation Method of Caffeine Consumption	Caffeine Exposure Timing	Main Outcome
Grodstein, 1993, USA * [27]	Case-control	N/A	2347	1666	N/A	180 women with confirmed endometriosis	3833 women who had been admitted for delivery to hospitals adjacent to the infertility clinics	Interview ^†^	Caffeine consumption	Positive association of endometriosis with caffeine consumption
Bérubé, 1998, Canada * [28]	Case-control	20–39	390	201	N/A	329 infertile women with minimal or mild endometriosis	262 infertile women without endometriosis	FFQ ^†^	Caffeine consumption	Positive association of endometriosis with >300 mg caffeine consumption
Pauwels, 2001, Belgium * [29]	Case-control	24–42	64	5	N/A	42 infertile women with confirmed endometriosis	27 infertile women without endometriosis	Interview ^†^	Caffeine consumption	No association of endometriosis with caffeine consumption
Parazzini, 2004, Italy * [30]	Case-control	20–65	619	389	N/A	504 women with confirmed endometriosis	504 women admitted to the hospital for acute non-gynaecological, non-hormonal, non-neoplastic conditions	FFQ ^††^	Caffeine consumption	No association of endometriosis with caffeine consumption
Missmer, 2004, USA [20]	Cohort	25–52	841	219	2 years	1721 women with confirmed endometriosis	N/A	FFQ ^†^	Caffeine consumption	No association of endometriosis with caffeine consumption
Buck Louis, 2007, USA * [21]	Case-control	27–37	57	27	N/A	32 women with confirmed endometriosis	52 women without endometriosis (after laparoscopy)	Interview ^††^	Caffeine consumption/ In utero exposure	Inverse association of endometriosis with in utero exposure to caffeine
De Oliveira, 2007, Brazil [31]	Case-control	15–45	95	22	N/A	39 women with histological confirmed abdominal scar endometriosis	78 with history of a previous obstetric hysterotomy without scar endometriosis	Questionnaire ^††^	Caffeine consumption	No association of scar endometriosis with caffeine consumption
Matalliotakis, 2008, USA * [22]	Cohort	15–47	110	26	6 years	535 women with confirmed endometriosis and pelvic pain or infertility	200 women without endometriosis (after laparoscopy)	Medical records ^††^	Caffeine consumption	No association of endometriosis with caffeine consumption
Huang, 2010, Taiwan * [32]	Case-control	27–45	22	35	N/A	28 women with confirmed endometriosis	29 women without endometriosis	Questionnaire ^††^	Caffeine consumption	No association of endometriosis with caffeine consumption
Wolff, 2013, USA [23]	Cohort	N/A	278	114	2 years	204 women diagnosed with endometriosis surgically or medically	396 women without endometriosis	Interview ^††^	In utero exposure	Inverse association of endometriosis with in utero exposure to caffeine
Saha, 2017, Sweden * [24]	Cross-sectional	20–65	21,573	7158	N/A	1228 women diagnosed with endometriosis surgically or medically	27,594 women without endometriosis	Interview ^†††^	Caffeine consumption	No association of endometriosis with caffeine consumption
Abadia, 2017, USA * [26]	Prospective cohort	29–40	220	93	8 years	14 infertile women with endometriosis	299 infertile women without endometriosis	FFQ ^†^	Caffeine consumption	N/A
Hemmert, 2019, USA * [25]	Prospective cohort	18–44	354	119	2 years	190 women Undergoing gynaecologic operation regardless of indication with endometriosis	283 women undergoing gynaecologic operation regardless of indication without endometriosis	Interview ^††^	Caffeine consumption	No association of endometriosis with caffeine consumption

N/A: Not Available, FFQ: Food Frequency Questionnaire. * Studies included in the meta-analysis, ** (>100 mg/day), *** (<100 mg/day). ^†^ Consumption in mg, ^††^ Consumption as yes/no, ^†††^ Consumption in cups.

**Table 2:** Risk of bias assessment of the included studies (MINORS tool).

Author, Year	Stated Aim	Consecutive Patients	Prospective Data Collection	Reported Endpoints	Unbiased Outcome Evaluation	Follow-Up Period Appropriate for the Study	Loss of Follow Up Less Than 5%	Adequate Control Group	Contemporary Groups	Group Matching	Prospective Sample Size Calculation	Adequate Statistics	Overall Score *	Risk of Bias **
Grodstein, 1993 [27]	2	0	0	2	0	0	0	1	0	0	0	2	7	High
Bérubé, 1998 [28]	1	0	0	2	0	0	0	1	2	1	0	2	9	High
Pauwels, 2001 [29]	1	0	0	1	1	0	0	1	0	1	0	2	7	High
Parazzini, 2004 [30]	1	0	2	2	0	0	0	2	0	2	0	1	10	Moderate
Buck Louis, 2007 [21]	1	0	2	2	1	2	0	2	0	1	0	1	12	Moderate
Matalliotakis, 2008 [22]	1	0	0	2	1	0	0	1	0	1	0	2	8	High
Huang, 2010 [32]	2	0	1	2	2	0	0	2	1	2	0	2	14	Moderate
Saha, 2017 [24]	2	1	2	2	1	0	0	2	1	0	0	2	13	Moderate
Abadia, 2017 [26]	1	0	1	1	1	0	0	2	0	1	0	2	9	High
Hemmert, 2019 [25]	1	0	1	2	1	0	1	1	1	2	2	2	14	Moderate

* The items were scored with 0 (not reported), 1 (reported inadequately), and 2 (reported adequately), ** Studies with a MINORS score of ≤9 were considered as high risk of bias, while studies scoring 10–14 were considered as moderate risk of bias.

## Data Availability

The data used to support the findings of this study are included within the article.
